# SQLE-mediated squalene metabolism promotes tumor immune evasion in pancreatic cancer

**DOI:** 10.3389/fimmu.2024.1512981

**Published:** 2024-12-23

**Authors:** Junchen Pan, Haixi Liang, Lin Zhou, Wenhua Lu, Bitao Huo, Rui Liu, Peng Huang

**Affiliations:** ^1^ Sun Yat-Sen University Cancer Center, State Key Laboratory of Oncology in South China, Collaborative Innovation Center for Cancer Medicine, Guangzhou, China; ^2^ Metabolic Innovation Center, Zhongshan School of Medicine, Sun Yat-sen University, Guangzhou, Guangdong, China

**Keywords:** SQLE, squalene, MDSCs, TAMs, NF-κB, pancreatic cancer

## Abstract

**Background:**

Squalene epoxidase (SQLE) is a key enzyme in cholesterol biosynthesis and has been shown to negatively affect tumor immunity and is associated with poor outcomes of immunotherapy in various cancers. While most research in this area has focused on the impact of cholesterol on immune functions, the influence of SQLE-mediated squalene metabolism within the tumor immune microenvironment (TIME) remains unexplored.

**Methods:**

We established an immune-competent mouse model (C57BL/6) bearing mouse pancreatic cancer xenografts (KPC cells) with or without stable SQLE-knockdown (SQLE-KD) to evaluate the impact of SQLE-mediated metabolism on pancreatic cancer growth and immune functions. The effect of squalene on tumor growth and immune cells was tested by direct administration of squalene to C57BL/6 mice bearing KPC tumors. Flow cytometry analysis and immunohistochemical (IHC) staining of immune cells from the tumor tissues were performed to evaluate changes in immune function. We also employed RNA-sequencing to analyze the gene expression profiles in pancreatic cancer cells (PANC-1) treated with or without squalene. RT-PCR and Western blot analyses were used to investigate the relevant molecular mechanisms.

**Results:**

We show that SQLE is significantly overexpressed in pancreatic cancer, and abrogation of SQLE results in a significant increase in squalene accumulation within tumor cells. The elevated squalene inhibits CXCL1 transcription through its impact on the NF-κB pathway via p65, and thus reduces the recruitment of myeloid-derived suppressor cells (MDSCs) and tumor-associated macrophages (TAMs) into the tumor microenvironment. Silencing of SQLE also leads to an increased proportion of CD8+ T cells in the tumor tissues and suppresses tumor growth *in vivo*. Importantly, direct administration of squalene, the metabolic substrate of SQLE, to immune-competent mice bearing KPC pancreatic cancer tumors causes a substantial decrease in CD206+ TAMs and MDSCs, thus releasing immune suppression and inhibiting tumor growth.

**Conclusion:**

Our study shows that squalene is an important immune-modulating metabolite that inhibits the infiltration of immune-suppressive cells in TIME, and that SQLE exerts its tumor immune evasion effect by metabolic removal of squalene. Thus, SQLE-mediated squalene metabolic pathway could be a potential target to enhance antitumor immunity in pancreatic cancer.

## Introduction

Pancreatic ductal adenocarcinoma (PDAC) is often not responsive to immunotherapy due in part to an immunologically “cold” tumor microenvironment, which contains immunosuppressive cells, including myeloid-derived suppressor cells (MDSCs) and tumor-associated macrophages (TAMs) (1). This tumor microenvironment typically shows a decrease in CD8+ T cell infiltration, resulting in a decrease of cytotoxic immune response (2–4). MDSCs have been found to infiltrate tumors, attracted by certain chemokines produced by the tumor cells (5). Their movement is primarily influenced by the chemokine receptors such as CXCR1/2 and their ligands including CXCL1, CXCL2, and CXCL5 (5). Macrophages within the tumor microenvironment, known as tumor-associated macrophages (TAMs), also play a major role in maintaining the immunosuppressive niche (6–8).

The tumor immune microenvironment (TIME) contains complex cellular components and is shaped by multiple factors such as hypoxia, nutrient availability, and other stress-related conditions, reflecting a complex interaction between tumor metabolism and immune responses via immunometabolic reprogramming (9–12). As a key enzyme in cholesterol biosynthesis, squalene epoxidase (SQLE) catalyzes the conversion of squalene to 2,3(S)-oxidosqualene (13). A recent study demonstrated that oxidative stress drives tumor progression through the activation of NR4A2-SQLE in microglia (14). The same study showed that pharmacological inhibition of NR4A2 could delay tumor development, while targeting either NR4A2 or SQLE enhances the effectiveness of immune checkpoint inhibitors in animal models (14). Abnormal SQLE activity disrupts cholesterol metabolism in microglia, fostering an immunosuppressive environment that supports glioblastoma (GBM) growth and advancement (14). In hepatocyte-specific SQLE knockout models, tumor suppression was observed, and this was associated with an increase in cytotoxic CD8+ T cells and a reduction in Arg-1+ MDSCs, suggesting a potential role of SQLE in maintaining the immunosuppressive environment in liver cancer (15). Interestingly, 24(S),25-epoxycholesterol (24(S),25-EC), a metabolite produced via the SQLE pathway, seems essential for the β-glucan-induced trained immunity in macrophages, which promotes antitumor effects (16). SQLE was also linked to tumor-infiltrating lymphocytes and immunomodulatory molecules, and was thought to be a promising biomarker for GBM prognosis and a potential target for glioma treatment (17). Knockdown of SQLE in melanoma models results in increased CD8+ T cell infiltration and reduced tumor growth, further supporting its potential as an immunotherapeutic target (18). Bioinformatic analyses also suggest that cholesterol metabolism mediated by SQLE overexpression correlates closely with infiltration of immune suppressive cells and a negative response to immunotherapy in pancreatic adenocarcinoma (PAAD) patients (17). Although SQLE’s role in TIME has been increasingly recognized, most studies attribute its effects to alterations in cholesterol metabolism. The role of squalene metabolism catalyzed by SQLE in affecting TIME and immunity against pancreatic cancer, however, remains unclear and requires further investigation.

In this study, we investigated the relationship between SQLE-driven squalene metabolism and immune regulation in PDAC, and found that SQLE-mediated metabolic removal of squalene played a major role in immune suppression in pancreatic cancer. We further showed that inhibition of SQLE led to a squalene-dependent reduction in the recruitment of immunosuppressive cells to the tumor microenvironment involving the CXCL1-mediated pathway. These results suggest that squalene metabolism governed by SQLE plays a previously unrecognized role in shaping the TIME.

## Materials and methods

### Cell lines

Human pancreatic cancer cell lines AsPC-1 and PANC-1 were obtained from the American Type Culture Collection (ATCC, Rockville, MD) and were maintained in 1640 and DMEM supplemented with 10% fetal bovine serum (FBS). The mouse pancreatic cancer KPC cells were maintained in DMEM supplemented with 10% FBS. All cell lines underwent regular testing to confirm their being free of mycoplasma contamination, using mycoplasma PCR detection (TaKaRa Taq Version 2.0, #R004).

### Mouse PDAC syngeneic model

Female C57BL/6 mice, aged 6 to 8 weeks, were utilized to create subcutaneous tumors of pancreatic cancer. KPC cells (1.8 × 10^6^ cells) were suspended in 100 μl PBS and injected subcutaneously into the flanks of the mice. Tumor volume was measured and calculated using the formula: volume = length × width² × 0.5. All animal experimental procedures adhered to the institutional guidelines and approved by the Animal Care and Use Committee of Sun Yat-sen University Cancer Center.

### RNA isolation and qRT-PCR

Total RNA was extracted from cultured cells or tumor tissues using the RNA-Quick Purification Kit (EZbioscience, B0004D). Complementary DNA (cDNA) was synthesized through reverse transcription of RNA with the Color Reverse Transcription Kit (A0010CGQ, Ezbioscience, USA). The relative levels of RNA were assessed by quantitative real-time PCR (qRT-PCR) utilizing the Bio-Rad detection system (Bio-Rad, Hercules, CA, USA). The expression levels of CXCL1 and NF-κB p65 were normalized by GAPDH using the comparative Ct method. The primers employed for qRT-PCR are as follows: CXCL1: AGCTTGCCTCAATCCTGCATCC, TCCTTCAGGAACAGCCACCAGT; NF-κB p65:ATGTGGAGATCATTGAGCAGC,CCTGGTCCTGTGTAGCCATT.

### Immunoblotting

Cells were lysed in RIPA buffer containing protease and phosphatase inhibitors (Cat No. P0013B, Beyotime Biotechnology, Shanghai, China). Protein samples were electrophoresed on 10%-15% polyacrylamide gels and transferred to PVDF membrane. Membranes were first incubated in blocking buffer (containing 5% dry milk powder) for 1 h at 24°C and then incubated with primary antibodies overnight at 4°C followed by secondary antibody (24°C, 2 h). Membranes were washed with PBST. Protein bands were detected by chemiluminescence using an ECL detection kit (Tanon, #180-5001). The following antibodies were used in this study: anti-SQLE (Proteintech, #12544-1-AP, dilution 1:1000), anti-CXCL1 (Proteintech, #12335-1-AP, dilution 1:1000), anti-GAPDH (Cell Signaling Technology, #14C10, dilution 1:1000), anti-NF-κB p65 (Cell Signaling Technology, #8242, dilution 1:1000), and anti-Phospho-NF-κB p65 (Ser536) (Proteintech, #80379-2-RR, dilution 1:1000).

### Flow cytometry analysis

Fresh tumors were minced and incubated in RPMI 1640 medium containing 0.1 mg/mL collagenase IV, 0.02 mg/ml DNase I, with 5% FBS at 37°C for 1 hour. The samples were then filtered through a 40 μm cell strainer (Corning) to prepare single-cell suspensions. Cell surface markers were stained with fluorophore-conjugated antibodies in the dark at 4°C for 45 minutes in Cell Staining Buffer (#420201, Biolegend). Flow cytometric analysis was performed using a CytoFLEX flow cytometer (Beckman Coulter). Cells stained with Zombie UV™ dye (#423107, Biolegend, dilution 1:1000), and alive cells were defined as Zombie UV-negative cells. Antibodies used in these analyses included FITC anti-mouse CD3ϵ (#100306, Biolegend, dilution 1:20), Pacific Blue anti-mouse CD4 (#100428, Biolegend, dilution 1:20), PE/Cyanine7 anti-mouse CD8a (#100722, Biolegend, dilution 1:20), APC/Cyanine7 anti-mouse CD45 (#103116, Biolegend, dilution 1:20), APC anti-mouse/human CD11b (#101211, Biolegend, dilution 1:20), Brilliant Violet 510 anti-mouse Ly-6G (#127633, Biolegend, dilution 1:20), Brilliant Violet 650 anti-mouse F4/80 (#123149, Biolegend, dilution 1:20), PE anti-mouse CD206 (#141706, Biolegend, dilution 1:20), Brilliant Violet 786 anti-mouse CD80 (#740888, BD OptiBuild, dilution 1:20). Up to 5×10^4^ CD45+ cells were recorded for further analysis by CytExpert.

### Immunohistochemistry analysis

Mouse tumor tissues were harvested, fixed in 4% formalin overnight, and subsequently embedded in paraffin. The paraffin-embedded tissues were sliced into 4μm-thick sections, and the endogenous peroxidase activity was quenched by incubation with 3% H_2_O_2_ for 15 minutes at room temperature. For antigen retrieval, the samples were boiled in an antigen-retrieval buffer (pH 8.0) for 5 minutes. After blocking with 3% BSA for 1 hour, the samples were then incubated overnight with the primary antibody at 4°C, and after washing, incubated with an HRP-conjugated secondary antibody for 1 hour at room temperature. DAB solution was then applied to visualize staining. The antibodies used were F4/80 (Proteintech, #28463-1-AP, IHC: 1:4000), CD206 (Invitrogen, #MR5D3, IHC: 1:200), and CXCL1 (Proteintech, #12335-1-AP, IHC: 1:75).

### RNA sequencing analysis

PANC-1 cells were treated with or without squalene for various time points as indicated, and the samples were subjected to RNA-seq analysis (Shanghai Majorbio Bio-Pharm Technology Co., Ltd) to identify differentially expressed genes (DEGs) in cells with or without squalene treatment. KEGG pathway enrichment analysis was performed to determine the pathways enriched with all differentially expressed genes. Fisher test was conducted to assess significantly enriched gene functions and KEGG pathways associated with DEGs. Enrichment analyses of KEGG pathways for DEGs were performed using the Python Scipy software package (https://scipy.org/install/). Pathways exhibiting a corrected p-value of less than 0.05 were considered significantly enriched.

### Analysis of gene expression datasets

Genotype-Tissue Expression (GTEx) (https://xenabrowser.net) and the Cancer Genome Atlas (TCGA) (https://www.cancer.gov/ccg/research/genome-sequencing/tcga) databases were used to obtain RNA-seq data for pancreatic cancer tissues (n=178) and normal pancreatic tissues (n=171). SQLE expression levels in the normal and tumor tissues were compared using the Wilcoxon rank-sum test.

### Statistical analyses

Statistical difference between two groups was assessed using the Student’s t-test. The relationship between two variables was evaluated using the Spearman’s correlation method. These statistical analyses were conducted with GraphPad Prism software (version 8.0, CA, USA). Gene expression levels in the normal and tumor tissues from public databases were compared using the Wilcoxon rank-sum test. A p-value of less than 0.05 was considered statistically significant. Data are expressed as mean ± S.D. as detailed in the figure legends.

## Results

### SQLE promotes pancreatic cancer growth *in vivo* via maintaining an immunosuppressive microenvironment

**Figure 1:**
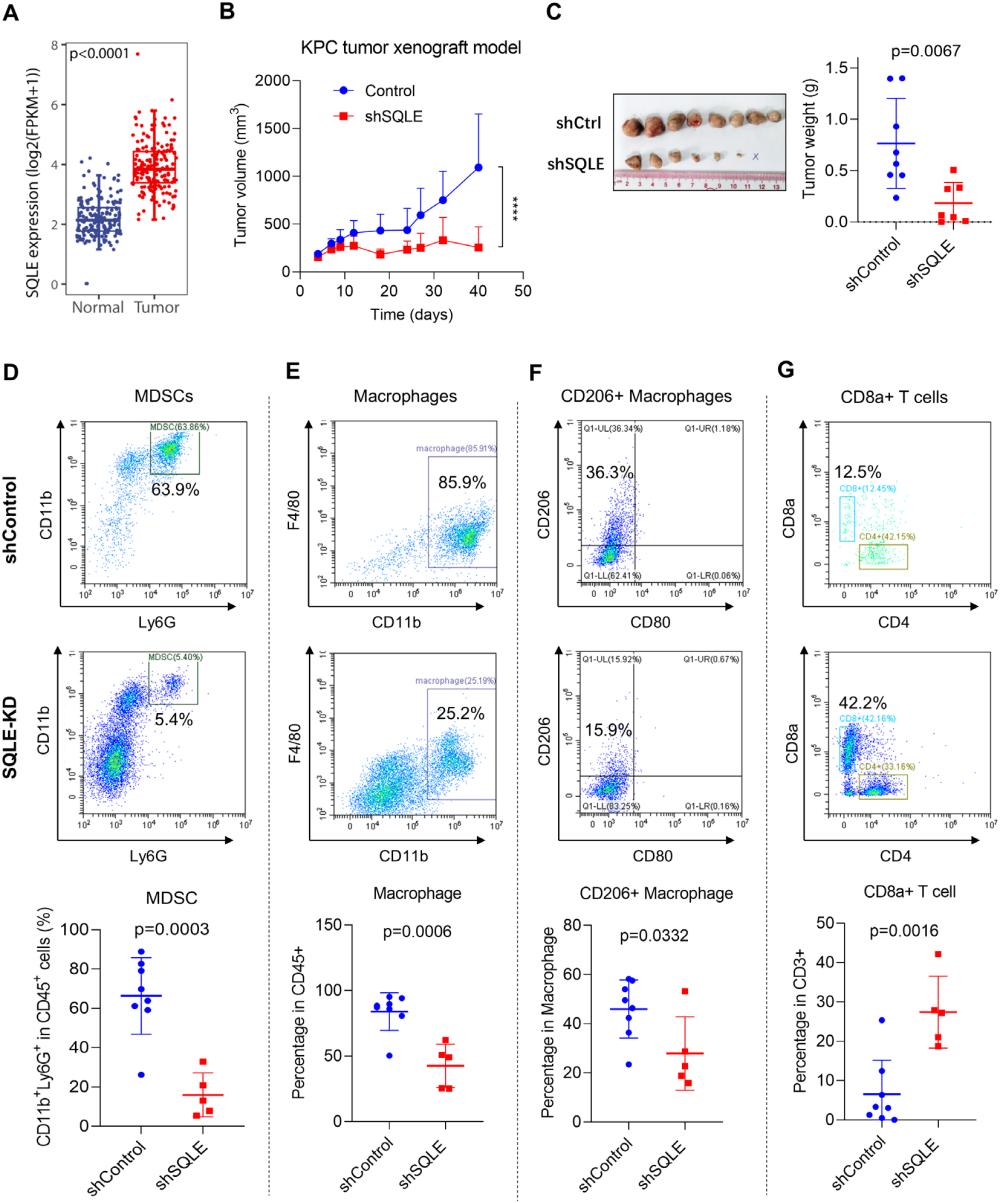
SQLE promotes *in vivo* tumor growth and elicits an immunosuppressive microenvironment in pancreatic cancer. **(A)** SQLE expression levels in pancreatic cancer and normal tissues. The comparison was performed using the GTEx and TCGA pancreatic datasets. **(B, C)** Impact of SQLE silencing by shRNA on tumor growth *in vivo*. C57BL/6 mice were injected with KPC cells containing either the control shRNA or specific SQLE-shRNA (1.8×10^6^ cells per injection). Measurements of tumor volume were shown in panel **(B)**. At the end of the experiment on day 40, tumors were isolated, photographed, and weighed **(C)**. The “x” symbol indicates a tumor disappeared in the SQLE-knockdown group. **(D–G)** Single-cell suspensions were prepared from the indicated tumor tissues. The proportions (%) of CD11b+Ly6G+ MDSCs **(D)**, CD11b+F4/80+ macrophages **(E)**, CD11b+F4/80+CD206+ macrophages **(F)**, and CD8+ T cells **(G)** in the gated subpopulations as indicated were analyzed using flow cytometry. The quantitative data of the specified cell subpopulations from all tumors of each group are shown in the bottom panels. Statistics: Wilcoxon rank-sum test **(A)**; Two-way ANOVA **(B)**; Unpaired student *t*-test **(C–G)**; ****, *p*< 0.0001.

To objectively evaluate the potential clinical relevance of SQLE expression, we first used public datasets from the GTEx and TCGA databases to compared the expression of SQLE in pancreatic cancer tissues (n=178) and normal tissues (n=171). Our analysis revealed that SQLE expression was significantly elevated in pancreatic tumor tissues (p<0.0001, **Figure 1A**). We then established an immune-competent mouse model (C57BL/6) bearing mouse pancreatic cancer tumors (KPC cells) with or without stable SQLE-knockdown (SQLE-KD) to evaluate the potential impact of SQLE-mediated metabolism on pancreatic cancer growth and immune functions. As shown in **Figures 1B, C**, shRNA-mediated silencing of SQLE expression led to a significant decrease in tumor growth compared to the control group. Some of the KPC cells with SQLE-KD were unable to form tumor in mice, while the control KPC cells were highly tumorigenic (**Figure 1C**). Since pancreatic cancer is immunologically “cold” tumor often infiltrated with immunosuppressive cells including tumor-associated macrophages and MDSCs consisted of aberrant monocytes and immature neutrophils (19), we then evaluated the impact of SQLE expression in pancreatic cancer cells on various immune cells in the tumor tissues. Flow cytometry analysis revealed that silencing of SQLE caused a major decrease in CD11b+/Ly6G+ MDSCs among the CD45+ leukocytes in the tumor tissues (**Figure 1D**). Quantitative analysis of multiple tumor tissues showed that over 60% of CD45+ cells were CD11b+/Ly6G+ MDSCs in the tumors of the control group, whereas the MDSCs decrease to less than 20% in the SQLE-KD group (**Figure 1D**, lower panel). There was also a significant decrease in CD11b+ macrophages in the SQLE-KD tumor tissues (**Figure 1E**). Interestingly, within the macrophage population, there was a significant decrease in CD206+ subpopulation (primarily consisting of M2 subtype) in the SQLE-KD tumor tissues (**Figure 1F**). Conversely, there was a substantial increase in CD8+ T cells in the SQLE-KD tumors (average 28%) compared to that in the shControl tumors (average 7%, **Figure 1G**). These data together suggest that SQLE might play an important role in promoting an immunosuppressive phenotype, and that its knockdown could mitigate such immunosuppression.

**Figure 2:**
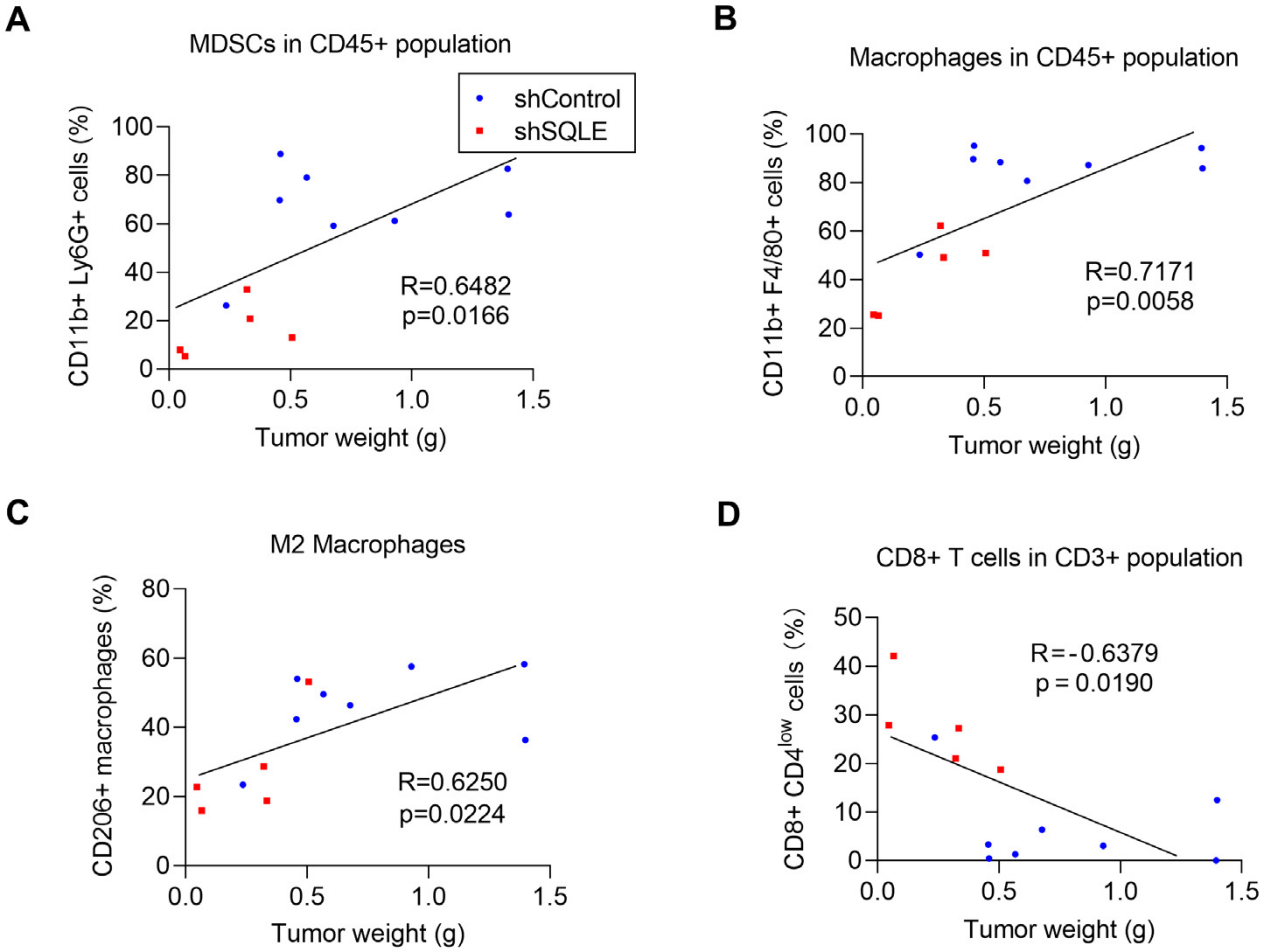
Correlation between degrees of immune cell infiltrations and tumor weights. The relationship between tumor weights and the percentages of infiltration of MDSCs in CD45+ subpopulation **(A)** macrophages in CD45+ subpopulation **(B)**, CD206+ macrophages **(C)** and CD8+ T cells in CD3+ subpopulation **(D)**. The blue dots indicate tumors inoculated with KPC cells transfected with control shRNA; The red dots indicate tumors inoculated with KPC cells transfected with specific shRNA against SQLE. Statistics: Linear regression **(A–D)**.

Consistently, analysis of the relationship between tumor sizes and immune cell infiltrations revealed a positive correlation between tumor sizes and the degrees of infiltrations of MDSCs and TAMs in the tumor tissues (**Figures 2A–C**). In contrast, there was a negative correlation between tumor sizes and CD8+ T cell infiltration, with larger tumors having fewer CD8+ T cells in the tumor microenvironment (**Figure 2D**). Interestingly, these correlations were observed when the data from the tumors of both groups (shControl and SQLE-KD) were pooled and plotted on the same charts, suggesting the critical role of the infiltrated immune cells in affecting the overall tumor growth. Of note, the SQLE-KD tumor samples contained less immunosuppressive cells (red dots in **Figures 2A–C**) and more cytotoxic CD8+ cells (red dots in **Figure 2D**), compared to that in the shControl tumors (blue dots).

### Squalene mediates SQLE-induced immunosuppression in pancreatic cancer

**Figure 3:**
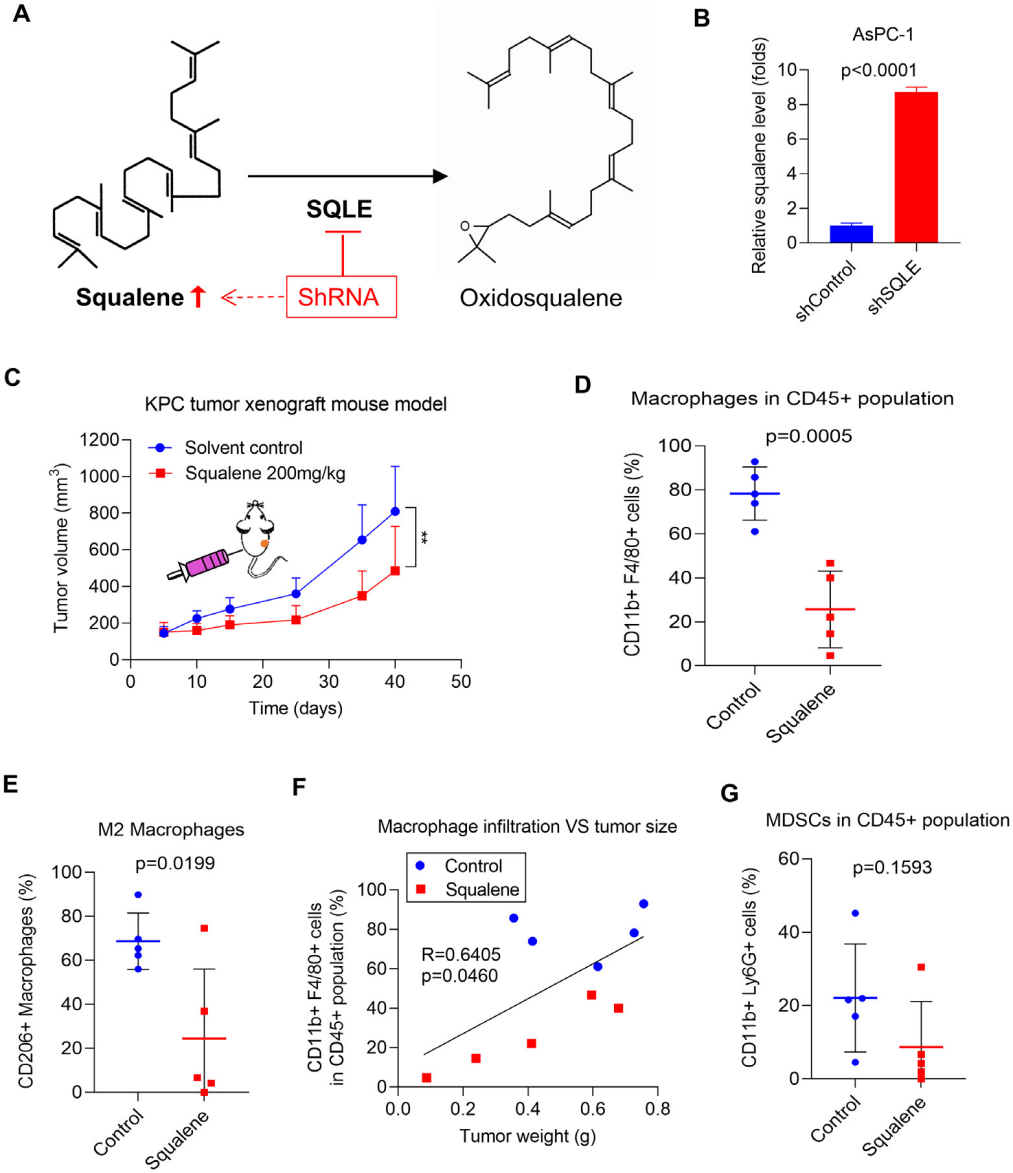
SQLE induces an immunosuppressive TME by metabolic removal of squalene’s effect on immune cell infiltration. **(A)** SQLE catalyzes the metabolic conversion squalene to oxidosqualene. **(B)** The levels of squalene in AsPC-1 cells transfected with control shRNA (shControl) or SQLE-specific shRNA (shSQLE). Squalene was analyzed using LC-MS and normalized by 1×10^6^ cells. **(C)** *In vivo* therapeutic effect of squalene (200 mg/kg every two days, i.p.) was evaluated in C57BL/6 mice bearing KPC tumors (1.8×10^6^ cells per injection; n=5 per group). Tumor sizes were measured and plotted as function of time. **(D, E)** The proportions of CD11b+F4/80+ macrophages and CD11b+F4/80+CD206+ macrophages in tumors from the indicated groups were measured using flow cytometry, and the quantitative data are shown as mean ± S.D. **(F)** Correlation between tumor weights and degrees (%) of macrophage infiltration. The blue dots indicate tumors from the control mice; The red dots indicate tumors inoculated tumors from squalene-treated mice. **(G)** The proportions of CD11b+Ly6G+ MDSCs in tumors from the indicated mouse groups were measured using flow cytometry, and the quantitative data are shown as mean ± S.D. Statistics: Unpaired student *t*-test **(B, D, E, G)**; Two-way ANOVA **(C)**; Linear regression **(F)**; **, *p*< 0.01.

Since SQLE is the key enzyme that catalyzes the conversion of squalene to oxidosqualene (13), we postulated that suppression of SQLE expression would cause an accumulation of squalene due to a stagnation of the metabolic flow (**Figure 3A**). We indeed observed that a stable knockdown of SQLE in pancreatic cancer cells (AsPC-1) by shRNA led to an 8-folds increase in cellular squalene (**Figure 3B**). Thus, we tested the effect of squalene on tumor growth and immune cells by direct administration of squalene to immunocompetent C57BL/6 mice bearing pancreatic cancer tumors (KPC cells). The results showed that *in vivo* treatment with squalene was able to significantly retard tumor growth in the KPC syngeneic model (**Figure 3C**). Flow cytometry analysis of immune cells from the tumor tissues revealed that squalene could reduce the percentage of macrophages (CD11b+ F4/80+) in the CD45+ cell population (**Figure 3D**) and also decreased the proportion of M2 (CD206+) macrophages (**Figure 3E**), similar to that observed in the SQLE-KD experiments. Consistently, the degrees of TAM infiltration appeared correlated with the tumor sizes, which were substantially smaller in the squalene-treated mice (**Figure 3F**, red dots) compared with the untreated mice (blue dots). Of note, the proportion of MDSCs (CD11b+ Ly6G+) in the CD45+ cell population in the tumor tissues showed a tendency of decrease after squalene treatment (**Figure 3G**), although such a reduction did not reach statistical significance likely due to the small sample size.

**Figure 4:**
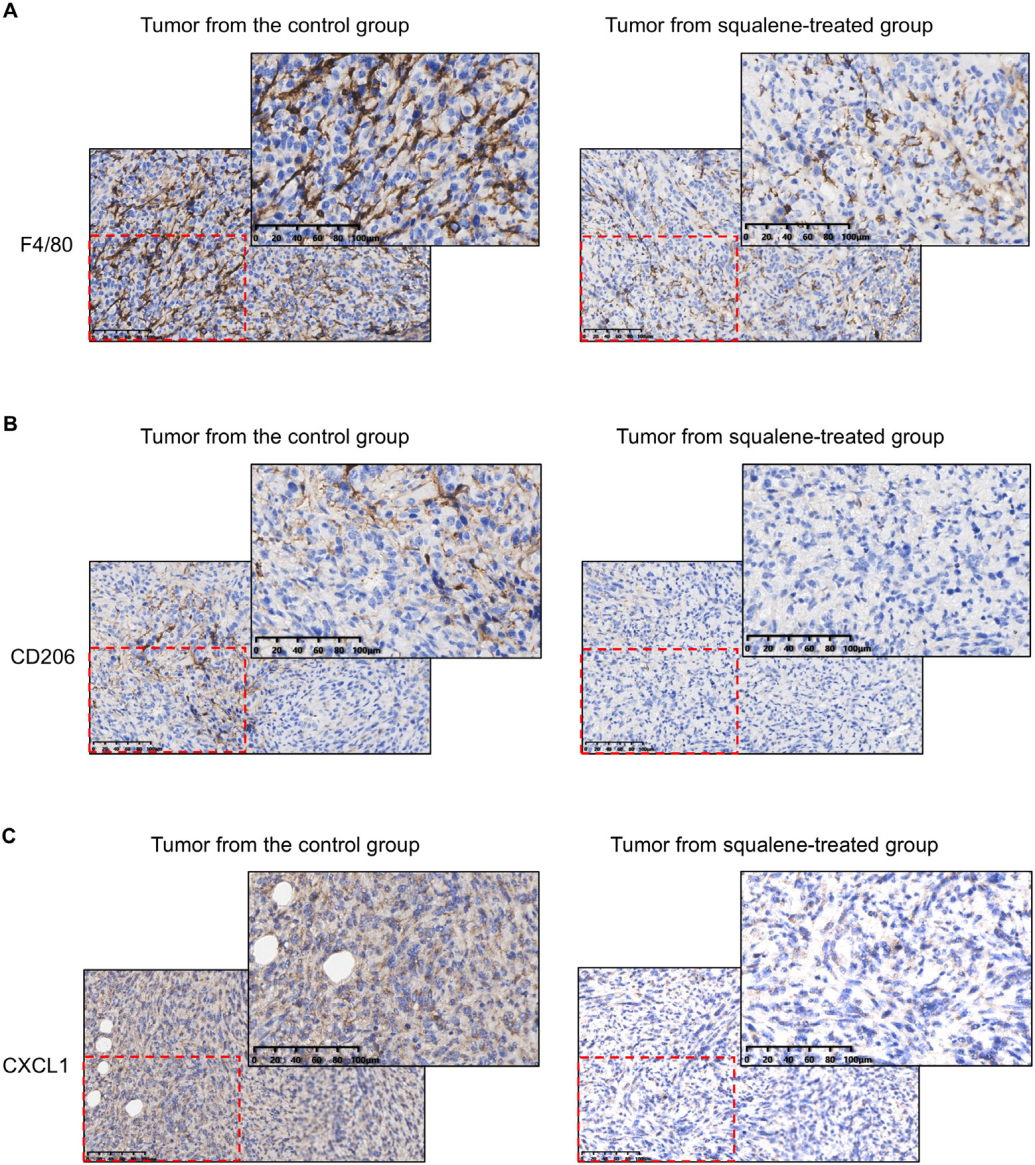
IHC staining for F4/80, CD206, CXCL1 in the tumor tissues from mice with or without squalene treatment. Representative images of IHC staining for F4/80 **(A)**, CD206 **(B)**, and CXCL1 **(C)** in tumor tissues from mice treated with or without squalene as indicated. The scale bar in each panel represents 100 µm.

The ability of squalene to inhibit the infiltration of TAMs was further confirmed by immunohistochemical (IHC) staining of the tumor tissues from the control mice and squalene-treated mice, as evidenced by a substantial decrease in F4/80+ cells (**Figure 4A**) and CD206+ macrophages (**Figure 4B**) in the squalene-treated group. These data (**Figures 3**, **4**) together showed that squalene was able to inhibit the infiltration of immunosuppressive cells into the tumor tissues, and suggest that it might likely mediate the immune-modulating effect observed in SQLE-KD tumor. Interestingly, we also observed that squalene treatment caused a substantial decrease in expression of CXCL1 (a chemokine known to recruit target cells such as MDSCs and TAMs (20–24)), in the tumor tissues (**Figure 4C**). These findings prompted us to further investigate the possibility that CXCL1 might be an important down-stream target of squalene as described below.

### Role of p65/NF-κB in the regulation of CXCL1 expression by SQLE/squalene

**Figure 5:**
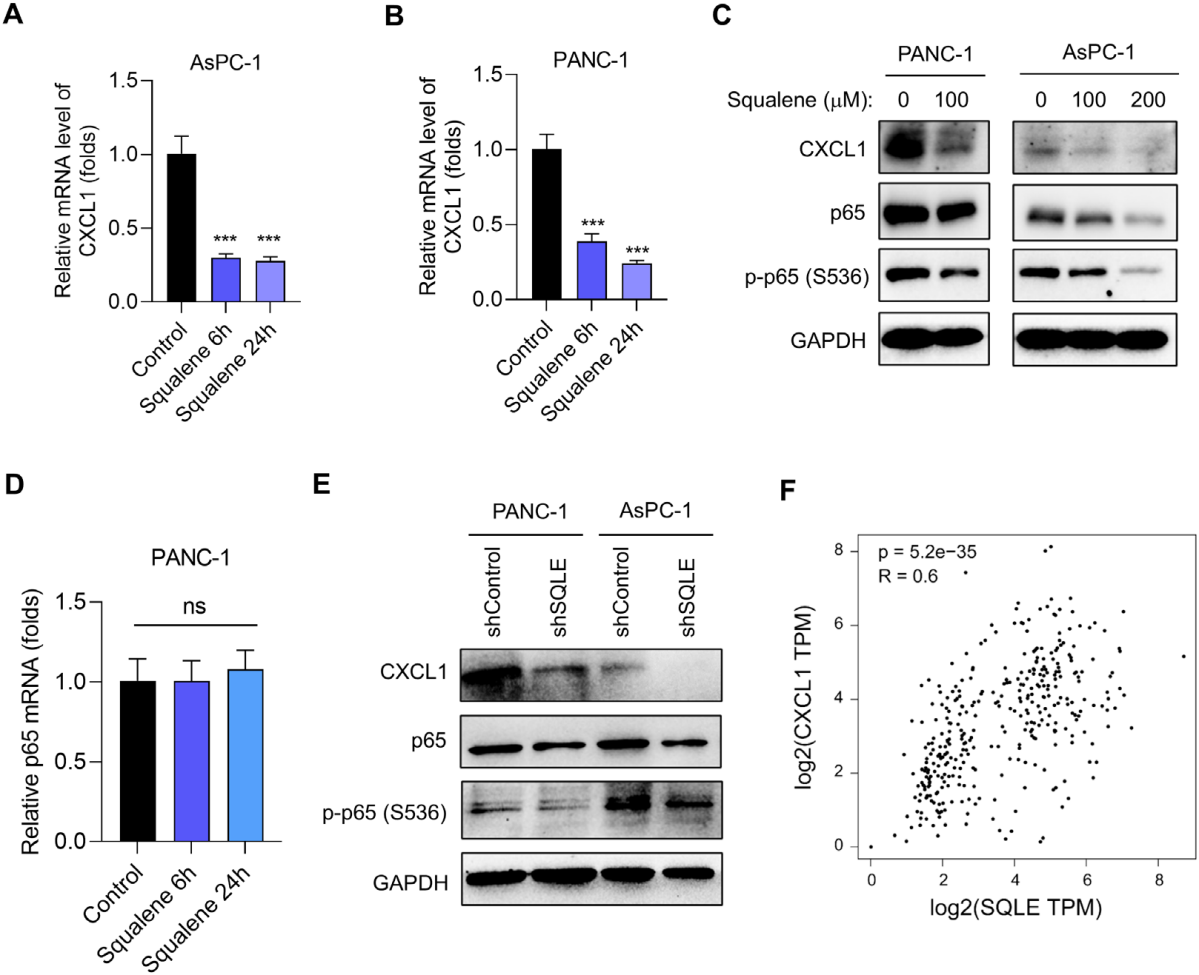
Suppression of SQLE leads to squalene-mediated inhibition of CXCL1 transcription. **(A, B)** Relative CXCL1 mRNA expression in AsPC-1 and PANC-1 cells treated with or without 200 μM squalene for 6 h and 24 h as indicated. **(C)** Western blot analysis was used to measure the expression of CXCL1, p65, and phosphorylated p65 (S536) in PANC-1 and AsPC-1 cells treated with the indicated concentrations of squalene for 24 hours. **(D)** The relative mRNA levels of p65 were measured in PANC-1 cells treated with 200 μM squalene for 6 h and 24 h as indicated. **(E)** Western blot analysis of the expression levels of CXCL1, p65, and phosphorylated p65 (S536) in PANC-1 and AsPC-1 cells transfected with either shControl or SQLE-targeting shRNA as indicated. **(F)** Relationship between SQLE and CXCL1 expression in pancreatic issues. TCGA datasets (normal & tumor) and GTEx (pancreas) dataset were analyzed using the Gene Expression Profiling Interactive Analysis (GEPIA) webtool. Statistics: One-way ANOVA **(A, B, D)**; ***, p< 0.001.

Since CXCL1 is a chemokine known to recruit target cells such as MDSCs and TAMs and contribute to an immunosuppressive microenvironment (20–25), we thus tested the possibility that squalene might affect CXCL1 expression and thus mediate the immune-modulating effect of SQLE. Quantitative RT-qPCR analysis showed that squalene could significantly inhibit the expression of CXCL1 mRNA in both AsPC-1 cells (**Figure 5A**) and PANC-1 cells (**Figure 5B**). Western blotting further demonstrated that squalene was able to substantially reduce CXCL1 protein the two pancreatic cell lines (**Figure 5C**). This Western blot analysis also revealed that treatment of pancreatic cells with squalene caused a decrease in the protein level of p65, a key component of the NF-κB signaling pathway known to regulate the expression of cytokines and chemokines including CXCL1 (26–28). The phosphorylation of p65 at S536 was also substantially reduced by squalene treatment (**Figure 5C**). These data suggest a possibility that squalene might regulate CXCL1 expression through the NF-κB pathway via affecting p65. Interestingly, RT-qPCR analysis showed that squalene did not cause any significant change in p65 mRNA level (**Figure 5D**), suggesting that squalene affected p65 at the post-transcriptional level.

Genetic silencing SQLE by shRNA, which caused squalene accumulation (**Figures 3A, B**), also led to an inhibition of CXCL1 expression consistently observed in both PANC-1 and AsPC-1 cells (**Figure 5E**). This was also associated with a decrease in p65 protein and a reduced phosphorylation at S536 (**Figure 5E**). These data were very similar to that observed with squalene treatment, and thus suggest that the effect of SQLE-KD on CXCL1 expression was likely mediated by the accumulation of its metabolic substrate (squalene). Of note, analysis of pancreatic cancer and normal tissue datasets from TCGA and GTEx databases revealed a positive correlation between SQLE and CXCL1 expression in clinical samples (**Figure 5F**), indicating that the regulation of CXCL1 by SQLE/Squalene likely occurred *in vivo*.

### RNA-sequencing characterization of cellular response to squalene

**Figure 6:**
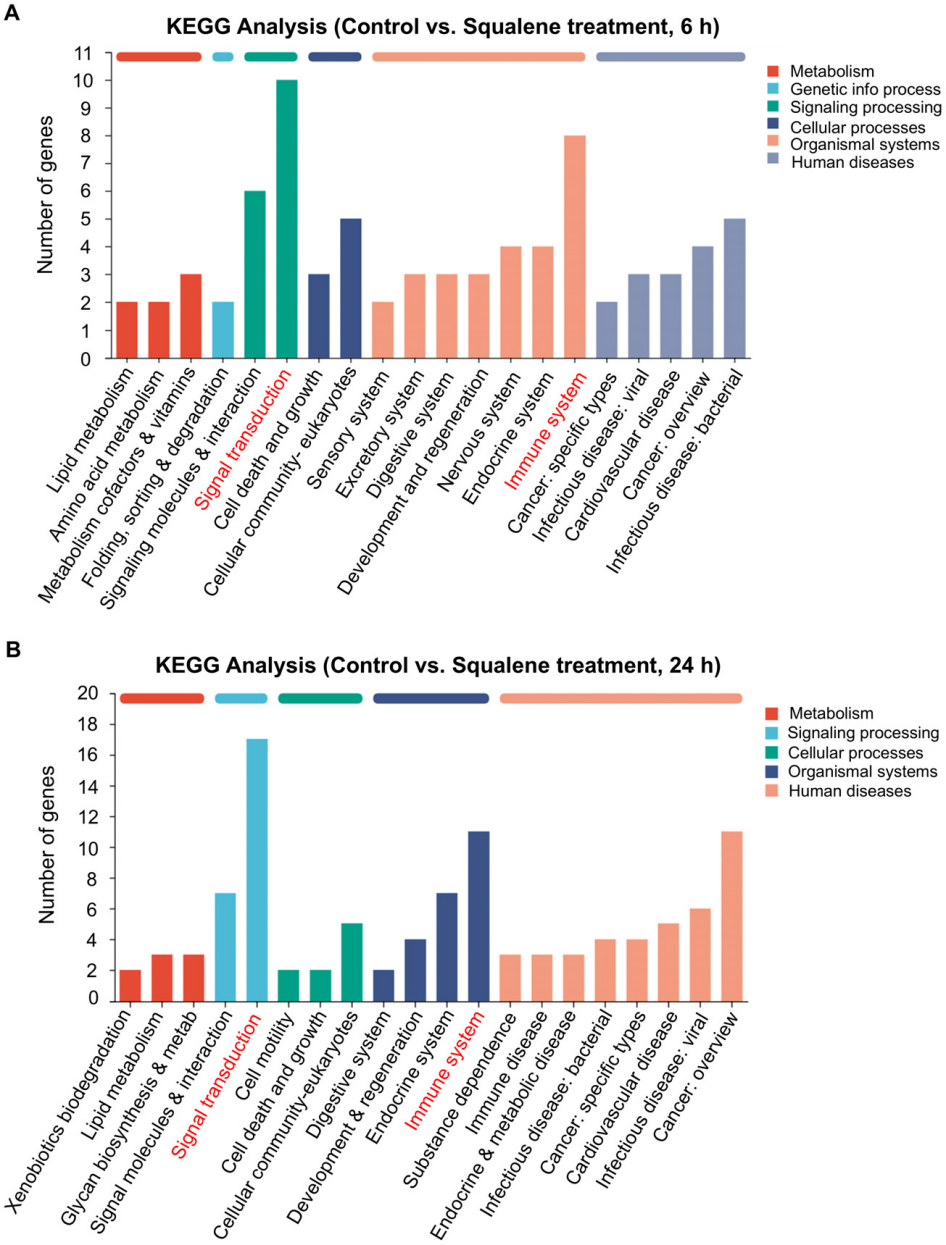
KEGG enrichment analysis of DEGs in PANC-1 cells treated with squalene for 6h or 24 h via RNA-seq. **(A)** Analysis of gene expression in PANC-1 cells with or without treatment with 200 μM squalene for 6 hours. Gene expression profiles were assessed through RNA sequencing. The differentially expressed genes (control *vs*. squalene-treated cells) in the indicated pathways or cellular processes were analyzed using the KEGG pathway analysis. **(B)** KEGG analysis of genes that were differentially expressed in PANC-1 cells after treatment with 200 μM squalene for 24 hours. Gene expression profiles were assessed and compared using the same methods as in **(A)**.

**Figure 7:**
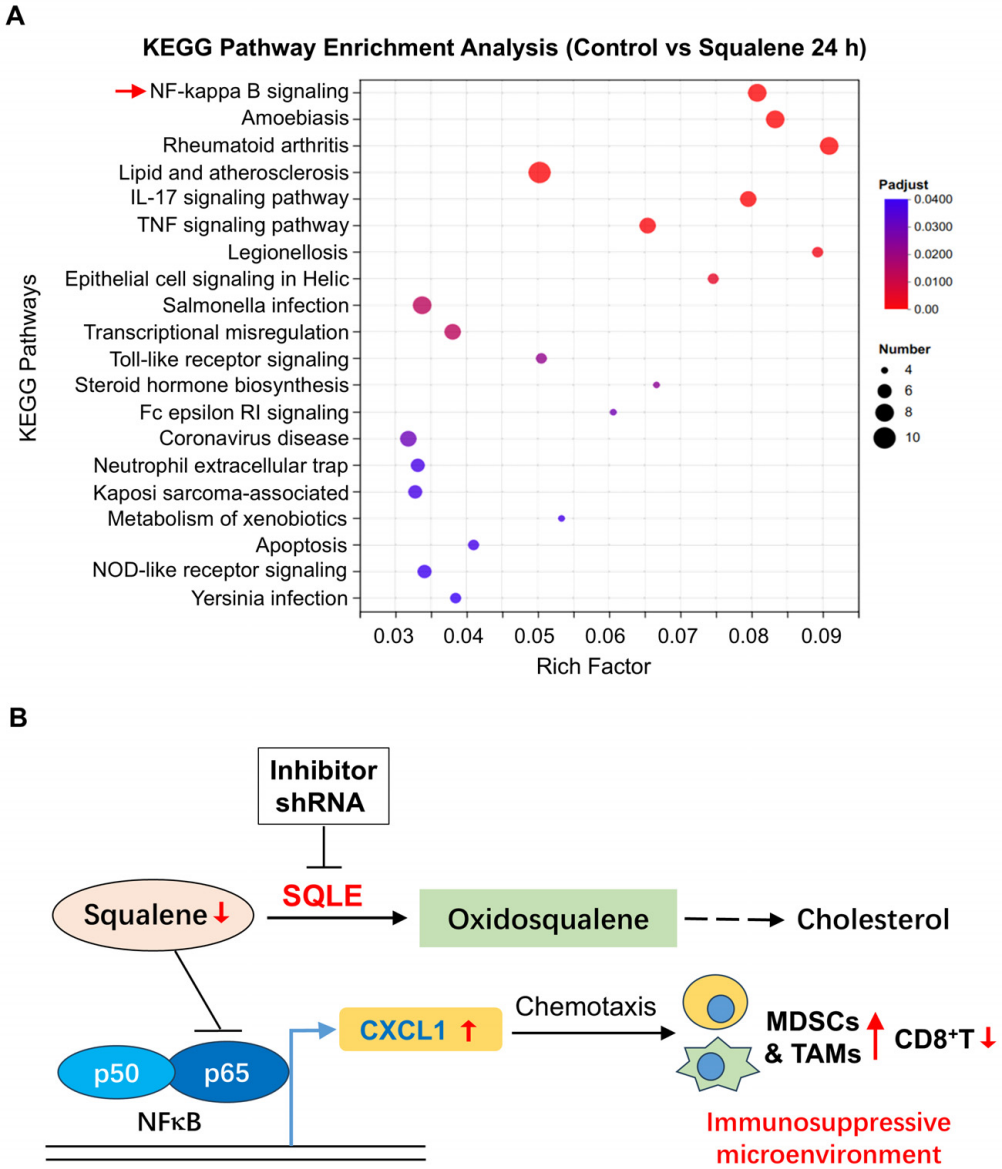
Effect of squalene on gene expression and immune functions. **(A)** Pathway enrichment analysis of genes that were differentially expressed in pancreatic cells with or without squalene treatment. PANC-1 cells were incubated with or without 200 μM squalene for 24 hours. Gene expression profiles were analyzed by RNA sequencing. The differentially expressed genes in various pathways and cellular processes were evaluated using the KEGG pathway enrichment analysis as described under Methods. **(B)** Schematic illustration of the role of SQLE and squalene in regulation of gene expression and immune functions. Squalene suppresses p65/NF-κB signaling pathway and thus suppresses the expression of CXCL1. In pancreatic cancer, SQLE (in red color) is upregulated and thus promotes the metabolic conversion of squalene to oxidosqualene, leading to a decrease of squalene (red arrow), less inhibition on NF-κB, and an increase in CXCL1 expression (red arrow). The chemotaxis effect of CXCL1 then attracts of myeloid-derived suppressor cells (MDSCs) and tumor-associated macrophages (TAMs), creating an immunosuppressive and pro-tumor microenvironment. Thus, the metabolic degradation of squalene by SQLE is important for tumor cells to maintain an immunosuppressive environment, and inhibition of SQLE would be a potential strategy to overcome immune evasion. Conversely, inhibition of SQLE by shRNA would lead to an accumulation of squalene, which suppresses NF-κB signaling and thus reduces the expression of CXCL1, leading to less infiltration of immunosuppressive TAMs/MDSCs and an increase of CD8+ T cells.

To further characterize the cellular processes in response to squalene treatment, we employed RNA-sequencing technology (RNA-Seq) to analyze the gene expression profiles in pancreatic cancer cells treated with or without squalene. PANC-1 cells were treated with 200 μM squalene for 6 and 24 h, and their gene expression profiles were characterized by RNA sequencing. Molecular analysis of the RNA-seq data using the Kyoto Encyclopedia of Genes and Genomes (KEGG) webtools revealed that squalene induced multiple changes in a variety of cellular processes, including metabolism, signal transduction, cellular processes, and other pathways (**Figure 6**). The squalene-induced changes in gene expression at the early time point (6 h, **Figure 6A**) and the next day (24 h, **Figure 6B**) were similar. Interestingly, “signal transduction” and “immune system” are among the pathway categories with most numbers of genes whose expression was altered (indicated by red color in **Figures 6A, B**). Genes related to “signal transduction” included FOS, RELB, SREBF1, MAP2K6, TLR2, TLR4, CSF2, CXCL1, CXCL8, CXCL3. Genes related to “immune system” were IFNE, LSP1, MAP2K6, TNFSF10, FCGR2A, CCR6, CCL20, CXCL1, CXCL8, CXCL3, and TLR4. Further characterization using KEGG pathway enrichment analysis showed that NF-κB signaling is the most prominent pathway with most changes induced by squalene (**Figure 7A**). The enriched genes in the NF-κB signaling pathways included TRAF1, RELB, GADD45A, TNFAIP3, TLR4, CXCL1, CXCL3 and CXCL8. These data were consistent with the Western blot data showing a decrease of p65 protein and inhibition of its phosphorylation by squalene (**Figure 5C**). Other pathways enriched in the squalene-treated cells included IL-17 signaling pathway, TNF signaling pathway, transcriptional misregulation, Toll-like receptor signaling, and neutrophil extracellular trap formation (**Figure 7A**), consistent with alterations of immune functions. Further reactome enrichment analyses revealed multiple pathways that were enriched in the squalene-treated cells (**Supplementary Figure S1A**). Among these enriched pathways, “signal transduction” pathways related to cytokine signaling and interleukin signaling in immune system were consistently identified (indicated by the red arrows in **Supplementary Figure S1A**). The names of the specific genes enriched in the relevant pathways are provided in **Supplementary Figure S1B**.

## Discussion

In this study, we first established a subcutaneous syngeneic mouse model bearing pancreatic cancer KPC cells with or without SQLE knockdown, and analyzed the changes of immune cells in the tumor tissues by flow cytometry. The significant findings of our study include the observations that silencing of SQLE significantly reduced the infiltration of tumor-associated macrophages and MDSCs in the tumor microenvironment, and the proportion of CD8+T cells was significantly increased. Considering that squalene is the metabolic substrate of SQLE, we tested the *in vivo* effect of squalene in the KPC syngeneic mouse model, and found that squalene treatment produced very similar effect on immune cells in the tumor microenvironment compared with the SQLE-knockdown model. These results suggest that squalene metabolism might play a major role in mediating the SQLE-induced immune suppression *in vivo* by affecting the infiltration of immunosuppressive cells in the tumor tissues.

A novel and significant finding from our study was the discovery that squalene could abrogate the immunosuppressive tumor microenvironment by inhibiting NF-κB-mediated expression of CXCL1, and thus significantly reduced its chemotaxis effect on the recruitment of immunosuppressive cells including MDSCs and TAMs. The mechanisms by which SQLE and squalene affect tumor immune functions are illustrated in **Figure 7B**. Under physiological conditions, squalene suppresses the p65/NF-κB signaling pathway and inhibits the expression of CXCL1. The decrease in CXCL1 has less ability to attract immunosuppressive cells such as MDSCs and TAMs, and thus maintaining an anti-tumor immune microenvironment. However, when SQLE expression is elevated in cancer such as in pancreatic ductal adenocarcinoma, its high enzyme activity could then promote the metabolic conversion of squalene to oxidosqualene, leading to a significant decrease of squalene and thus less inhibition on NF-κB signaling pathway, which in turn promotes CXCL1 expression to recruit more MDSCs and TAMs to the tumor tissues, and thus creating an immunosuppressive and pro-tumor microenvironment. The metabolic clearance of squalene by SQLE is an important biochemical mechanism for the tumor cells to maintain an immunosuppressive tissue microenvironment. As such, inhibition of SQLE by specific shRNA or small chemicals would be a potential strategy to overcome cancer immune evasion.

Since NF-κB signaling pathway is known to regulate the expression of CXCL1 (29, 30), which in turn promotes chemotaxis of MDSCs and TAMs through CXCR2 receptor (31–33), our data together suggest that SQLE likely affects cancer immunity through metabolic elimination of squalene, leading to activation p65/NF-κB signaling pathway and high expression of CXCL1 to exert its chemotaxis effect on MDSCs and TAMs, which promote the formation of an immunosuppressive tumor microenvironment. However, the mechanism by which squalene downregulates p65 protein still remains unclear. Our data suggest that such a downregulation likely occurred at post-transcriptional level, since squalene caused a decrease in p65 protein without altering p65 mRNA expression (**Figures 5C, D**). Further studies are required to explore the underlying mechanisms. Of note, the CD11b+/Ly6G+ MDSCs identified by flow cytometry analysis in our study (**Figure 1D**) represented the polymorphonuclear subtype of MDSCs (PMN-MDSCs), since CD11b+ and Ly6G+ are considered markers of PMN-MDSCs (20, 34, 35). It would be interesting to utilize additional markers such as Ly6C and CD49d to evaluate the effect of SQLE and squalene on the monocytic subtype of MDSCs (M-MDSCs).

There have been multiple reports on the impact of SQLE on the tumor microenvironment and immune functions. However, most of these studies mainly focused on the role of SQLE-catalyzed production of cholesterol in affecting tumor cells and immune cells (15, 18). In contrast, our study demonstrated that squalene, the metabolic substrate of SQLE, played a key role in mediating the effect of SQLE on immune cells. This novel conclusion is supported by multiple lines of evidence. (1) Abrogation of SQLE by shRNA caused a major increase in squalene accumulation in the cancer cells; (2) Direct administration of squalene to mice bearing tumors caused changes of immune cells in the tumor tissues very similar to the changes of immune cells in the tumor with SQLE knockdown; (3) Treatment of cancer cells with squalene *in vitro* led to inhibition the p65/NF-κB signaling pathway and a decrease of CXCL1 expression, which was also observed in cells with SQLE knockdown.

In summary, our study demonstrated that squalene is a negative regulator of p65/NF-κB signaling pathway and suppresses the expression of CXCL1. The metabolic removal of squalene by SQLE causes the activation of NF-κB pathway and promote the expression of CXCL1, leading to elevated infiltration of immunosuppressive cells in the tumor tissues to facilitate tumor growth. These new findings provide novel insights into the relationship between squalene metabolism and tumor immunity.

## Data Availability

The datasets presented in this study can be found in online repositories. The names of the repository/repositories and accession number(s) can be found below: NCBI, accession number, PRJNA1174173 (https://www.ncbi.nlm.nih.gov/sra/PRJNA1174173).

## Supplementary material

Supplementary Figure 1 Related genes in the KEGG enrichment pathway were listed according to RNA-sequencing results. **(A)** PANC-1 cells were incubated with or without 200 μM squalene for 24 hours. Gene expression profiles were analyzed by RNA sequencing. Reactome enrichment analysis was then used to reveal genes that were differentially expressed in pancreatic cells treated with or without squalene treatment. The red arrows indicate the signal transduction pathways related to cytokine/interleukin signaling in immune system; **(B)** List of specific genes differentially expressed in pancreatic cells treated with or without squalene. These genes were identified by KEGG pathway enrichment analysis.
