# Supplementary figures and images for: SQLE-mediated squalene metabolism promotes tumor immune evasion in pancreatic cancer
Source: Front Immunol. 2024 Dec 23;15:1512981. doi: 10.3389/fimmu.2024.1512981 (PMC11701373; doi:10.3389/fimmu.2024.1512981)

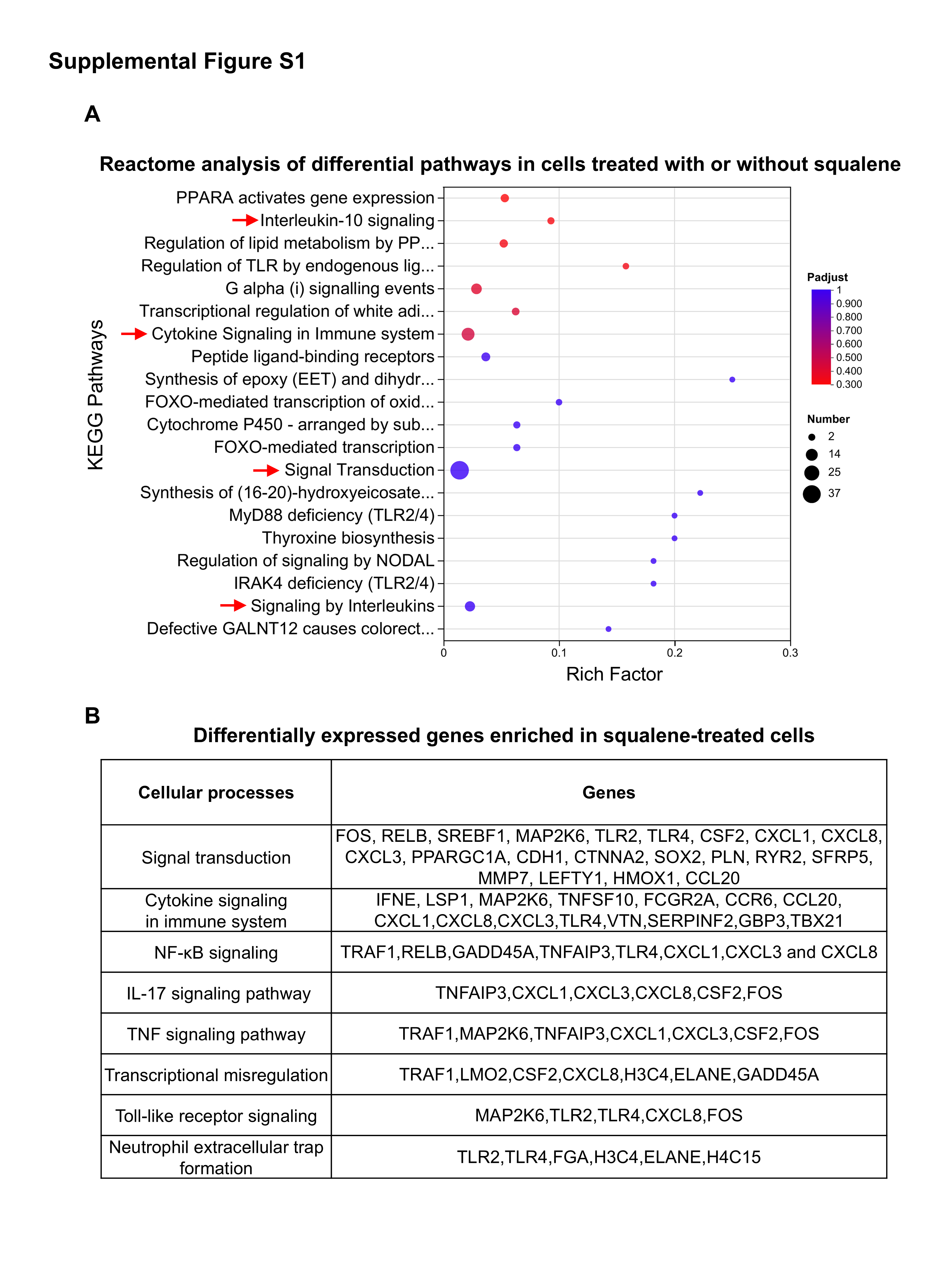

Supplement: Supplementary Figure 1 — Related genes in the KEGG enrichment pathway were listed according to RNA-sequencing results. (A) PANC-1 cells were incubated with or without 200 μM squalene for 24 hours. Gene expression profiles were analyzed by RNA sequencing. Reactome enrichment analysis was then used to reveal genes that were differentially expressed in pancreatic cells treated with or without squalene treatment. The red arrows indicate the signal transduction pathways related to cytokine/interleukin signaling in immune system; (B) List of specific genes differentially expressed in pancreatic cells treated with or without squalene. These genes were identified by KEGG pathway enrichment analysis. [file Image1.tif]
